# The Life of Brian … and David: Two Flying‐Foxes' Contribution to Science

**DOI:** 10.1002/ece3.72834

**Published:** 2026-01-02

**Authors:** M. J. Walker, J. A. Welbergen, J. Meade, W. S. J. Boardman, T. Reardon, J. M. Martin, A. McKeown, C. Turbill

**Affiliations:** ^1^ Hawkesbury Institute for the Environment Western Sydney University Richmond New South Wales Australia; ^2^ School of Life and Environmental Sciences, Faculty of Science, Engineering and Built Environment Deakin University Burwood Victoria Australia; ^3^ Department of Pathobiology and Population Health, School of Animal and Veterinary Sciences University of Adelaide Roseworthy South Australia Australia; ^4^ Cudlee Creek South Australia Australia; ^5^ Commonwealth Scientific and Industrial Research Organisation (CSIRO) Land and Water Urrbrae South Australia Australia; ^6^ School of Science Western Sydney University Richmond New South Wales Australia

**Keywords:** banding, biotelemetry, field procedures, surgery, welfare, wildlife

## Abstract

The welfare of free‐living animals in scientific studies must always be in question: do procedures and devices deployed on wildlife negatively affect their welfare? In December 2018, we captured two wild, male grey‐headed flying‐fox ( *Pteropus poliocephalus* ), given the names Brian and David, at Adelaide Botanic Park, South Australia. Brian and David, two of nine bats captured, were estimated to be 3 and 5 years old. We tagged the bats with thumb bands and surgically implanted a transmitter to monitor body temperature. Following release, body temperature data were collected (21 and 65 days, respectively). Brian was found deceased in June 2025, 6.5 years later, 2.6 km from the capture site. David was found deceased in April 2024, 5.3 years later, 9.4 km from the capture site. A necropsy was performed on each bat. This research note addresses the question: do thumb bands and transmitter implantation negatively affect the welfare of flying‐foxes? Through Brian and David's case studies, detailing the procedures, physiological data and necropsy findings, we present observations to suggest that, in these individuals, these procedures and devices had negligible impacts. While the case of two individuals offers limited power for broader inference, long‐term post‐procedure data of telemetry‐equipped animals is rare, making these observations valuable to report. We thank these bats' involuntary but valuable contribution to science and present this research note to celebrate the life of Brian and David.

## Graphical abstract

This Nature Note examines whether thumb bands and surgically implanted temperature transmitters adversely affect the welfare of free‐living grey‐headed flying‐foxes. Long‐term monitoring and necropsy findings from two males, Brian and David, suggest negligible impacts of these procedures on individual welfare. Although based on only two cases, the rare long‐term data provide valuable insight into post‐procedure outcomes for telemetry‐equipped wildlife.

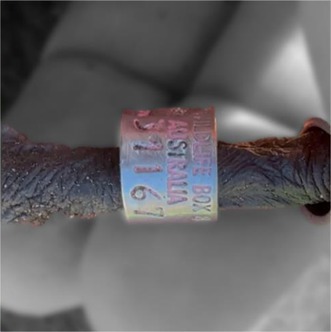

## 1 Background

Biotelemetry has advanced our understanding of how free‐living animals behaviourally and physiologically respond to the environment. These data are particularly valuable for assessing a species' ability to cope with environmental fluctuations and other ecological challenges (Whitford and Klimley 2019; Cooke et al. 2004), and biologging is increasingly positioned to support such assessments by enabling more direct investigation of animal welfare in natural settings (Beaulieu and Masilkova 2024). For cryptic or highly mobile species, biologging devices offer critical insights, overcoming observational limitations and filling essential knowledge gaps to support effective conservation, management strategies and animal welfare assessments (Brown et al. 2013). For example, core body temperature monitoring has revealed that free‐living grey‐headed flying‐foxes, *Pteropus poliocephalus* , experiencing days of extreme heat, exhibit controlled hyperthermia to reduce evaporative water loss (Walker et al. 2025) and, in cold, wet, and windy conditions, use torpor to conserve energy (Turbill et al. 2024). Further, GPS tracking has demonstrated these nocturnal bats are not only highly mobile (Welbergen et al. 2020), but have some capacity for navigation and orientation when displaced from their colony (Meade et al. 2025), and in urban landscapes, use both native and exotic food resources (Yabsley et al. 2022). These studies suggest a degree of behavioural flexibility that may buffer these bats against environmental change, an important consideration for assessing their welfare in the face of future climatic extremes and modified landscapes.

Insight from these free‐living animals is made possible by capturing individuals and equipping them with data logging or transmitting devices. Such procedures, particularly when applied to a species for the first time, require ethical justification and a clear understanding of their potential impact on animal welfare. However, long‐term data on the impacts of these techniques remain scarce due to the difficulty of monitoring highly mobile animals over extended periods, which can complicate the assessment of applications for approval by animal care and ethics committees and limit methodological developments. This scarcity partly reflects complex ethical considerations unique to free‐living animals (Lindsjö et al. 2016). Accordingly, when the opportunity arises to assess the welfare implications of such procedures, it is important to report them. Doing so supports future research by providing evidence of any welfare impacts, which can inform ethical approvals and guide improvements to procedures where necessary. In this research note, we present a case study of free‐living flying‐foxes monitored by biotelemetry, highlighting the seemingly negligible impact on life following the procedures that included deploying thumb bands and a surgically implanted device.

## 2 Animal Capture and Device Deployment

Wild bats were captured at the Adelaide flying‐fox camp, at Botanic Park, South Australia, on 4 December 2018. Processing, attachment of identification bands, and transmitter implantation were undertaken at veterinary facilities at the nearby Adelaide Zoo. All procedures were conducted once the individual had been placed on a heated mat (3 M Bair Hugger Normothermia System, Canada), anaesthetised by inhalation via a face mask of isoflurane (5% induction, 2%–3% maintenance) in oxygen (1.5 L min^−1^), and given a non‐steroidal anti‐inflammatory drug for pain relief (Carprofen 3 mg/kg, Rimadyl, Zoetis Inc., Kalamazoo, MI) and 20–40 mL of Hartmann's fluid to counter any dehydration. Initial processing included a condition assessment and collection of morphometric data: for Brian, these were a 4/5 body condition based on sternal protrusion representing above average body condition, a mass of 663 g, a forearm length of 166.1 mm and an estimated age of 3 years based on testes development and molar wear (Divljan et al. 2006); for David, these were a 2.5/5 body condition, a mass of 799 g, a forearm length of 173.8 mm and an estimated age of 5 years.

**FIGURE 1:**
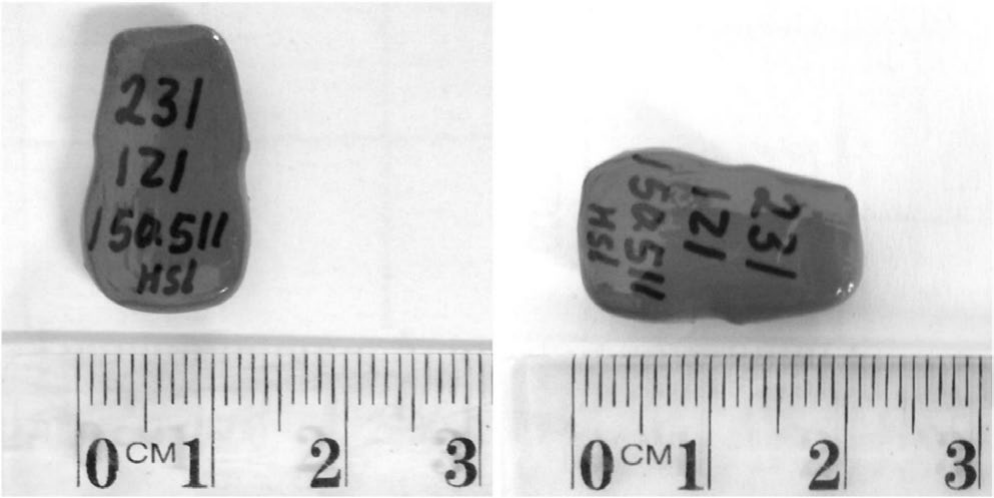
An example of the temperature‐sensitive VHF FM radio (model PD‐2THX) implanted into Brian and David, free‐living grey‐headed flying‐foxes.

Following the initial assessment, bats were fitted with thumb bands, and a temperature‐sensitive VHF FM radio transmitter was surgically implanted. Four stainless‐steel bands, one uniquely numbered and coloured and three coloured, obtained through the Australian Bird and Bat Banding Scheme (ABBBS licence number 9180) were used for identification. The band number used on Brian was 083‐31167 and the colour combination on left and right thumbs was KYKB (pink, yellow; pink, blue) and for David 083‐31005 and KYRW (pink, yellow; red, white). The temperature‐sensitive transmitter (model PD‐2THX, Holohil, Ontario, Canada, 3.9 g; Figure 1) was used to measure core body temperature (for more detail, see Walker et al. 2025). Data received from this device could be collected manually using a handheld receiver (R410 model, Advanced Telemetry Systems) or autonomously using two programmed data‐logging receivers (R4500S model, Advanced Telemetry Systems). Brian was assigned a unique transmitter (serial number: 231126), with a frequency of 150.850, and David 231123, 150.650. For implantation, a 4 by 2 cm midventral patch of hair was shaved and aseptically prepared with iodine and 70% ethanol under sterile conditions. A 2 cm incision along the ventral midline allowed insertion of the transmitter, with muscle and skin closed separately using absorbable sutures (Vicryl Plus 4‐0, Ethicon Inc., Somerville, NJ). The implanted transmitter represented 0.6% (Brian) and 0.5% (David) of body mass. Following these procedures, 100% oxygen was provided until recovery from anaesthesia (< 2 min), and following assessment by a veterinarian (WB), bats were released back into the Adelaide camp.

## 3 Data Obtained

**FIGURE 2:**
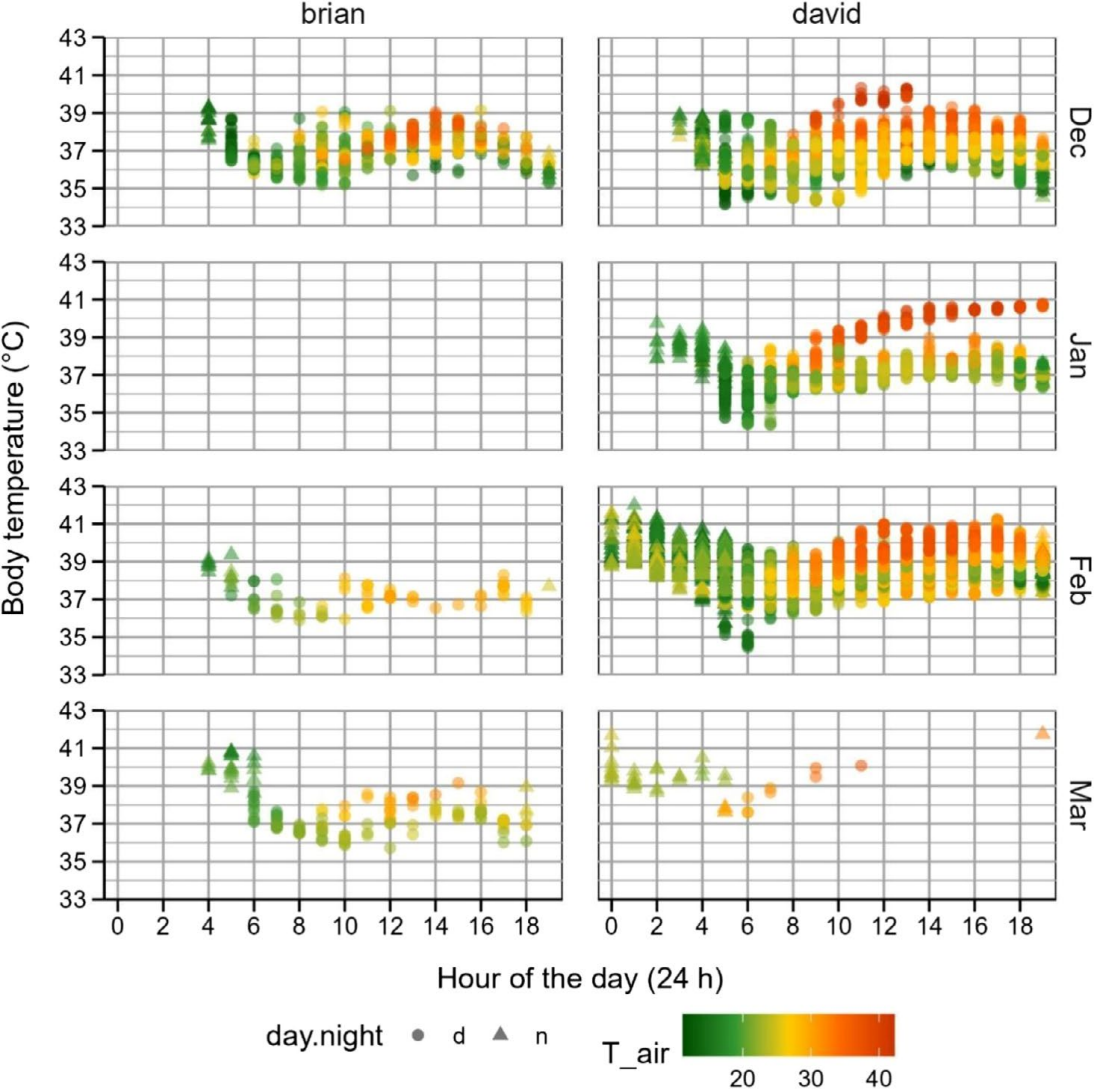
Body temperature (°C) data from Brian and David, two free‐living grey‐headed flying‐foxes, plotted as a function of hour of the day (day = circles, night = triangles), by month, and coloured by air temperature (°C). Data were recorded between December 2018 and March 2019.

Over 105 days between 8 December 2018 and 23 March 2019, 21 days of body temperature data were collected from Brian and 65 days were collected for David. Brians limited days of data were explained by him being within range of an autonomous logger for only 20% of the post‐release monitoring period, and never within range of the handheld receivers. In total, data were recorded on 34 days in December, 16 in January, 23 in February and four in March. Body temperature varied with hour of the day and air temperature (Figure 2; sensu Walker et al. 2025), and while at the camp during the day, bats maintained an average (±SE) body temperature of 37.3°C ± 0.3°C (*N* = 7540), ranging from an absolute minimum of 34.2°C to an absolute maximum of 41.3°C. At night, the average was slightly higher, at 38.9°C ± 0.1°C (*N* = 1258, range: 34.5°C–42.0°C).

## 4 Results of the Necropsy

Flying‐foxes demonstrate low fidelity to camp sites and are highly mobile; the maximum movement recorded for a *P. poliocephalus* individual, for example, was 12,337 km across 123 camps over 1629 tracking days (~4.5 years, Welbergen et al. 2020). While the Adelaide flying‐fox camp is currently the only major camp in South Australia, there are numerous smaller camps around the greater Adelaide region (Australasian Bat Society 2025). However, 6 years after their capture, Brian's body was found in the suburb of Fitzroy (13 June 2025, 34.896° S, 138.590° E), only 2.6 km from the camp, while David's body was found in the suburb of Valley View (21 April 2024, 134.838° S, 138.649° E), only 9.4 km away from the camp. The bodies were stored in −20°C freezer until necropsies were performed on the thawed carcasses on 15 June 2025 (Brian) and 31 Aug 2025 (David) by WB.

Banding and implantation procedures appeared to have had no adverse long‐term impact on health or body condition and did not contribute to the death of either bat. The necropsy of Brian showed evidence of free blood in both the pleural and peritoneal cavities, as well as a discrete laceration 4 mm long on the mid cortical region of the right kidney. The right dactylopatagium showed a recent tear along the leading edge, and there were puncture wounds and bruising over the dorsal thoracic and lumbar regions. These injuries were consistent with predation, and the suspected cause of death was an attack, most likely by a raptor because of the lack of observed fractures that would suggest a larger predator such as a fox, dog or cat. Records from Bird Life Australia (2025) indicate that 13 raptor species occur in the greater Adelaide region, including eagles, harriers, hawks, kites and owls. Of these, only the Barn Owl ( *Tyto alba* ) and Southern Boobook ( *Ninox boobook* ) are nocturnal, but to our knowledge, there are no reports of either species preying on *P. poliocephalus* , though predation by other owls in other regions has been documented (e.g., Powerful owl ( *Ninox strenua* ), Mo et al. 2025). Diurnal predators known to be present in the Fitzroy area include the Peregrine falcon ( *Falco peregrinus* ) which normally hunts at dusk and dawn but is also known to hunt at night particularly in cities. Other diurnal raptors, the White‐bellied Sea‐Eagle (*Icthyophaga leucogaster*) and Wedge‐tailed Eagle ( *Aquila audax* ), reported to prey on *P. poliocephalus* (Ratcliffe 1932; Nelson 1965) are not seen in this area. It remains unclear whether Brian was predated during the day while roosting away from camp or at night while foraging. The necropsy of David showed evidence of electrocution. The right ear was singed to the base of the skull, and the tongue tip was black and dry. The trailing edge of the right dactylopatagium major, dactylopatagium medius and the plagiopatagium was shredded and dry. Electrocution is a global threat to bats (O'shea et al. 2016), and Australian flying‐foxes are no exception (Preece 2025). Death by electrocution may occur when bats collide with utility wires (O'shea et al. 2016) or come into contact with electrified crop nets (Martin 2011). Given that David was found in a suburban location, it is likely that he was electrocuted by utility wires.

At the time of necropsy, the body condition of both bats was good. Brian's forearm measured 178.4 mm, an increase of 12.3 mm over 6 years, and mass was 942 g, 279 g more than at the time of capture. The teeth were worn but in good condition, with all four canines, incisors and molars present. Based on molar wear (Divljan et al. 2006), Brian was approximately 9.5 years old at the time of death, which is consistent with the assessment of age at time of capture 6.5 years before his death. David's forearm measured 173.8 mm, the same as at capture, and mass was 873 g, 74 g more than at the time of capture. The incisors were worn, which is not surprising given that David was approximately 12 years old at the time of death. The age of both bats falls above the average, and, in David's case, well above the range of lifespan observed for banded grey‐headed flying‐foxes, with 7.1 ± 3.9 years previously reported by Tidemann and Nelson (2011).

**FIGURE 3:**
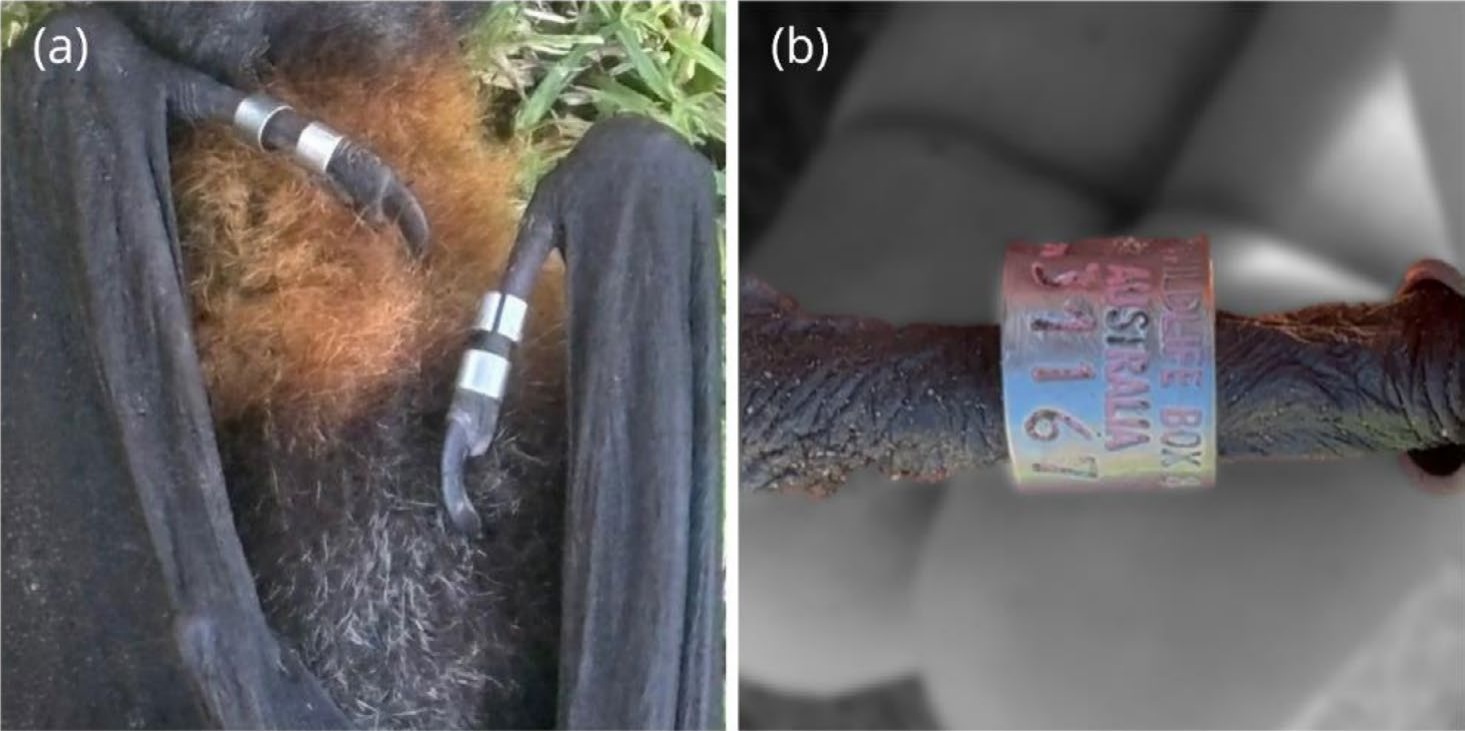
Thumb bands remained in place for over 6 years and were examined when Brian, a free‐living grey‐headed flying‐fox, was found deceased. All bands were intact (a), and identification numbers remained legible (b). However, colour had largely faded. Brian's band number was 083‐31167, with original left and right thumb colours KYKB (pink, yellow; pink, blue).

All four thumb bands on both bats remained intact, and the band number was still legible, though little to no colour could be identified on any band (Figure 3). There was no evidence of banding‐related injuries. Each of the temperature‐sensitive transmitters was located in the left cranial peritoneal cavity. There were no signs of adhesions, indicating that the material used for sealing the transmitter did not stimulate an immune reaction. It is clear that the equipment used to collect data does not appear to have impacted the life of either animal.

## Author Contributions

**M. J. Walker:** conceptualization (equal), data curation (equal), formal analysis (equal), visualization (equal), writing – original draft (equal). **J. A. Welbergen:** conceptualization (equal), funding acquisition (equal), supervision (equal), writing – review and editing (equal). **J. Meade:** conceptualization (equal), supervision (equal), writing – review and editing (equal). **W. S. J. Boardman:** methodology (equal), writing – original draft (equal). **T. Reardon:** methodology (equal), writing – review and editing (equal). **J. M. Martin:** methodology (equal), writing – review and editing (equal). **A. McKeown:** methodology (equal), writing – review and editing (equal). **C. Turbill:** conceptualization (equal), funding acquisition (equal), supervision (equal), writing – review and editing (equal).

## Funding

This research was funded by an Australian Research Council Discovery Project awarded to Welbergen and Turbill (DP170104272).

## Ethics Statement

This research was conducted under a Scientific Research Permit from the South Australian Government (M26735‐3) and an Animal Research Authority approved by the Western Sydney University Animal Care and Ethics Committee (A12217).

## Conflicts of Interest

The authors declare no conflicts of interest.

## Data Availability

Body temperature data are available in DRYAD. These data were previously published in association with Walker et al. (2025), DOI: https://doi.org/10.5061/dryad.djh9w0w92.
